# Co-infection of COVID-19 and influenza A in a hemodialysis patient: a case report

**DOI:** 10.1186/s12879-020-05723-y

**Published:** 2021-01-13

**Authors:** Ran Jing, Rama Rao Vunnam, Elizabeth Schnaubelt, Chad Vokoun, Allison Cushman-Vokoun, David Goldner, Srinivas Rao Vunnam

**Affiliations:** 1 Department of Internal Medicine, University of Nebraska College of Medicine, 985520 Nebraska Medical Center, Omaha, NE 68198 USA; 2 Penn State College of Medicine, 700 HMC Crescent Road, Hershey, PA 17033 USA

**Keywords:** Case report, COVID-19, Co-infection, Influenza A, High risk, Hemodialysis

## Abstract

**Background:**

Coronavirus disease 2019 (COVID-19) is caused by the severe acute respiratory syndrome coronavirus 2 (SARS-CoV-2), a novel coronavirus that was first discovered in December 2019 in Wuhan, China. With the growing numbers of community spread cases worldwide, the World Health Organization (WHO) declared the COVID-19 outbreak as a pandemic on March 11, 2020. Like influenza viruses, SARS-CoV-2 is thought to be mainly transmitted by droplets and direct contact, and COVID-19 has a similar disease presentation to influenza. Here we present a case of influenza A and COVID-19 co-infection in a 60-year-old man with end-stage renal disease (ESRD) on hemodialysis.

**Case presentation:**

A 60-year-old man with ESRD on hemodialysis presented for worsening cough, shortness of breath, and diarrhea. The patient first developed a mild fever (37.8 °C) during hemodialysis 3 days prior to presentation and has been experiencing worsening flu-like symptoms, including fever of up to 38.6 °C, non-productive cough, generalized abdominal pain, nausea, vomiting, and liquid green diarrhea. He lives alone at home with no known sick contacts and denies any recent travel or visits to healthcare facilities other than the local dialysis center. Rapid flu test was positive for influenza A. Procalcitonin was elevated at 5.21 ng/mL with a normal white blood cell (WBC) count. Computed tomography (CT) chest demonstrated multifocal areas of consolidation and extensive mediastinal and hilar adenopathy concerning for pneumonia. He was admitted to the biocontainment unit of Nebraska Medicine for concerns of possible COVID-19 and was started on oseltamivir for influenza and vancomycin/cefepime for the probable bacterial cause of his pneumonia and diarrhea. Gastrointestinal (GI) pathogen panel and *Clostridioides difficile* toxin assay were negative. On the second day of admission, initial nasopharyngeal swab came back positive for SARS-CoV-2 by real-time reverse-transcriptase polymerase chain reaction (RT-PCR). The patient received supportive care and resumed bedside hemodialysis in strict isolation, and eventually fully recovered from COVID-19.

**Conclusions:**

We presented a case of co-infection of influenza and SARS-CoV-2 in a hemodialysis patient. The possibility of SARS-CoV-2 co-infection should not be overlooked even when other viruses including influenza can explain the clinical symptoms, especially in high-risk patients.

## Background

Coronavirus disease 2019 (COVID-19), a novel coronavirus disease that was first reported in Wuhan, China, is caused by the severe acute respiratory syndrome coronavirus 2 (SARS-CoV-2) [1]. With the growing numbers of community spread cases worldwide, the World Health Organization (WHO) declared the COVID-19 outbreak as a pandemic on March 11, 2020 [2]. As of June 29, 2020, over 10 million cases have been reported in 188 countries, causing more than 500,000 deaths [3]. The first COVID-19 case in the United States (US) was confirmed on January 20, 2020, and in a mere 5 months, the widespread community transmission of the virus has led the number of confirmed cases in the US to exceed 2 million, and the death toll has surpassed 126,000 as of June 29, 2020 [3, 4]. Like influenza viruses, SARS-CoV-2 is thought to be mainly transmitted via respiratory droplets or direct contact, and COVID-19 has a similar disease presentation to influenza, causing respiratory diseases that present as a broad-spectrum of illnesses from asymptomatic or mild to severe illness and, in some cases, death [5, 6]. Here we present a case of COVID-19 and Influenza A co-infection in a patient on hemodialysis who has no travel history or known exposure to suspected or confirmed COVID-19 cases in the United States.

## Case presentation

A 60-year-old man with end-stage renal disease (ESRD) on hemodialysis via arteriovenous (AV) fistula presented to the emergency department (ED) from home on March 18, 2020, with a chief complaint of fever, cough, shortness of breath, and diarrhea. He has a history of IgA nephropathy and a failed kidney transplant due to rejection in 2013. He lives alone at home in western Iowa and had no recent travel history or visits to any healthcare facilities except for a local dialysis center for routine dialysis.

The patient receives hemodialysis three times a week (Monday, Wednesday, and Friday) at a local dialysis center. The symptoms started on Monday, March 16, 2020, while he was at his routine dialysis when he began to feel feverish and experience chills. His temperature taken at the dialysis center was 37.8 °C. After dialysis, he developed a headache, non-productive cough, sore throat, runny nose, poor appetite with nausea, vomiting, and abdominal pain, which progressively worsened over the next 2 days. On March 17, he started to have generalized myalgias, mild shortness of breath, and diarrhea with liquid green stool without visible blood. He missed his hemodialysis on Wednesday, March 18 and presented to the ED.

**Fig. 1:**
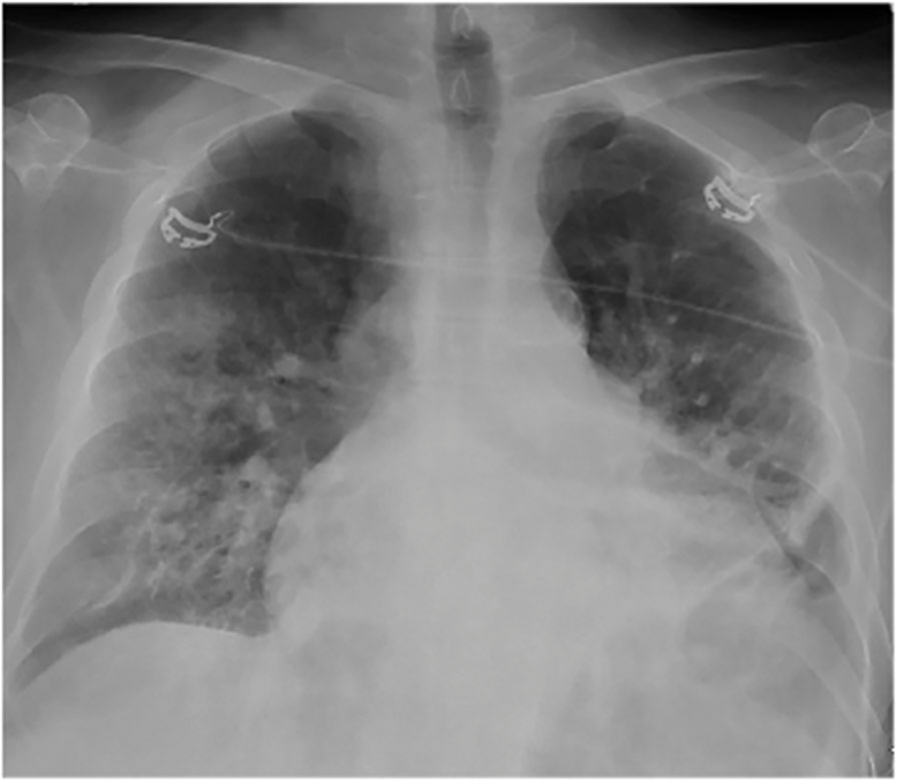
Anteroposterior chest X-ray showing new right midlung consolidation and left lower lung opacities

On arrival at the ED, the patient was provided a mask and placed in a private room in accordance with the ED screening protocol. He was ill-appearing with a fever of 38.6 °C. His respiratory rate was 23 per minute, and other vital signs were unremarkable. Physical examination was significant for diffuse rhonchi and bibasilar rales on auscultation. Chest X-ray (Fig. 1) showed right midlung consolidation, and patchy left lower lung opacities, concerning for multifocal pneumonia. Rapid influenza antigen test was positive for influenza A; viral subtypes were not specified. A nasopharyngeal swab specimen was sent for real-time reverse-transcriptase polymerase chain reaction (RT-PCR) test (NEcov19 assay) at the Nebraska Medical Center Clinical Lab to rule out COVID-19 infection. He was placed on contact and airborne isolation precautions with eye protection for concerns of probable COVID-19 as per the Centers for Disease Control and Prevention (CDC) recommendations.

**Fig. 2:**
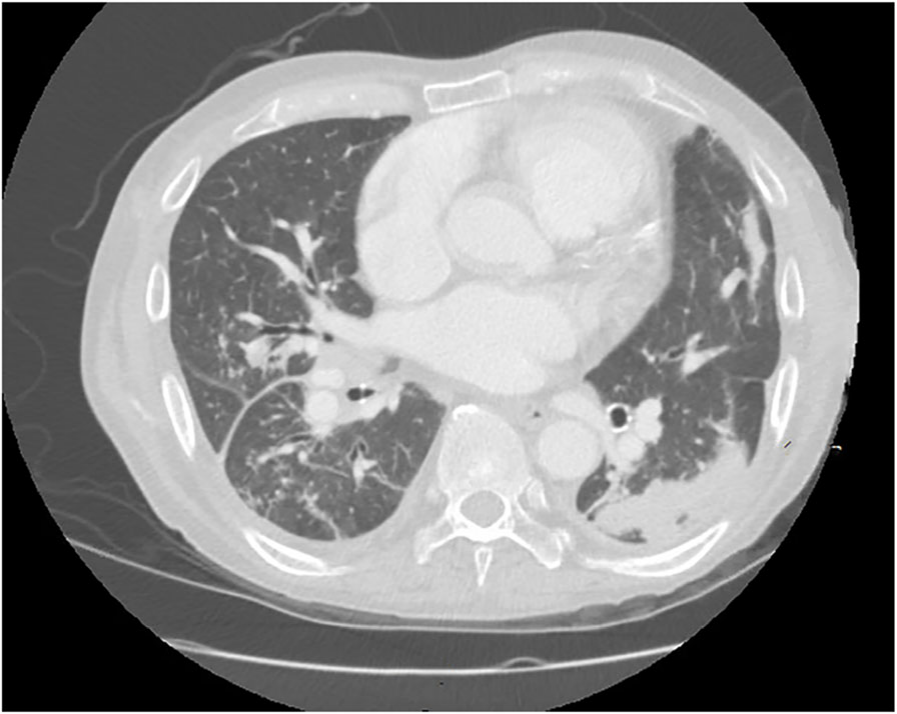
CT chest with no contrast showing multifocal pneumonia

Lab workup was significant for elevated procalcitonin and lymphopenia (Table 1). The electrocardiogram showed normal sinus rhythm. Computed tomography (CT) of the chest (Fig. 2) demonstrated multifocal areas of consolidation and tree-in-bud opacities within multiple lobes of the lungs compatible with multifocal pneumonia, as well as small bilateral pleural effusions and extensive mediastinal and bilateral hilar adenopathy. CT abdomen and pelvis showed mildly dilated gas and fluid-filled loops of small bowel and bowel wall enhancement, reflecting possible partial distal obstruction or ileus and enteritis. Blood cultures were taken, and the patient was started on oseltamivir (30 mg, oral, once per day on Mon Wed Fri, given after hemodialysis) for influenza. He is anuric; therefore, we did not test urine streptococcal and legionella antigen. The patient was then admitted to a negative pressure room on an isolation ward while awaiting COVID-19 results.

**Table 1:** Laboratory results

Laboratory Measures	Reference Range	Hospital Day 1 (Illness Day 3)	Hospital Day 2 (Illness Day 4)	Hospital Day 3 (Illness Day 5)	Hospital Day 4 (Illness Day 6)	Hospital Day 5 (Illness Day 7)	Hospital Day 6 (Illness Day 8)
White Cell Count (per μl)	4000–11,000	8700	─	15,300 (H)	7400	6100	6800
Red Cell Count (per μl)	4,300,000 – 5,900,000	3,660,000(L)	─	3,190,000 (L)	3,400,000 (L)	3,270,000 (L)	3,400,000 (L)
Hemoglobin (g/dL)	13.3–17.3	11.3 (L)	─	9.9 (L)	10.4 (L)	10.2 (L)	10.4 (L)
Hematocrit (%)	39.0–52.0	33.5 (L)	─	30.1 (L)	31.8 (L)	31.0 (L)	32.1 (L)
Platelet Count (per μl)	150,000 – 400,000	226,000	─	157,000	179,000	189,000	199,000
Absolute Neutrophil Count (per μl)	1300–7500	7200	─	13,300 (H)	5500	3800	4700
Absolute Lymphocytes Count (per μl)	700–3900	800	─	1200	1200	1500	1300
Prothrombin Time (sec)	10.1–14.2	13.3	14.5 (H)	14.2	─	─	─
International Normalized Ratio	0.9–1.1	1.1	1.2 (H)	1.2 (H)	─	─	─
Sodium (mmol/L)	136–145	130 (L)	130 (L)	128 (L)	131 (L)	─	─
Potassium (mmol/L)	3.5–5.1	5.4 (H)	5.1	5.1	3.7	─	─
Chloride (mmol/L)	98–107	91 (L)	96 (L)	96 (L)	96 (L)	─	─
Anion Gap (mmol/L)	4–15	18 (H)	14	16 (H)	11	─	─
CO2 (mmol/L)	22–32	21 (L)	20 (L)	16 (L)	24	─	─
Blood Urea Nitrogen (mg/dL)	6–20	57 (H)	58 (H)	64 (H)	26 (H)	─	─
Creatinine (mg/dL)	0.64–1.27	8.42 (H)	9.63 (H)	10.90 (H)	6.68 (H)	─	─
Glucose (mg/dL)	70–139	53 (L)	245 (H)	97	69 (L)	─	─
Magnesium (mg/dL)	1.8–2.5	─	2.0	2.2	1.9	─	─
Calcium (mg/dL)	8.6–10.4	9.1	7.9 (L)	8.6	8.5 (L)	─	─
Total Protein (g/dL)	5.8–8.2	6.4	─	5.0 (L)	5.5 (L)	5.6 (L)	─
Albumin (g/dL)	3.5–5.1	3.0 (L)	─	2.3 (L)	2.6 (L)	2.5 (L)	─
Aspartate Aminotransferase (U/L)	15–41	95 (H)	─	66 (H)	68 (H)	60 (H)	─
Alanine Aminotransferase (U/L)	7–52	55 (H)	─	40	36	36	─
Alkaline Phosphatase (U/L)	32–91	104 (H)	─	111 (H)	92 (H)	91	─
Conjugated Bilirubin (mg/dL)	0.1–0.5	─	─	0.1	0.1	0.2	─
Total Bilirubin (mg/dL)	0.3–1.0	0.7	─	0.5	0.5	0.5	─
Lactic Acid (mmol/L)	0.5–2.0	1.3	─	0.8	─	─	─
Procalcitonin (ng/mL)	< 0.10	5.21 (H)	─	17.30 (H)	15.30 (H)	─	10.00 (H)

(L) The value in the patient was below normal

(H) The value in the patient was above normal

On hospital day 2, his RT-PCR test was positive for SARS-CoV-2 E and/or N gene RNA, confirming the diagnosis of COVID-19. The local health department was notified to investigate the source of infection. The patient reported feeling extremely fatigued but was afebrile. His cough and shortness of breath improved. His primary complaint was severe liquid green diarrhea, although the Gastrointestinal (GI) pathogen panel and *Clostridioides difficile* (C. diff) toxin assay were negative. The repeated procalcitonin level (Table 1) was elevated on day 2, suggestive of potentially fivefold increased risk of severe disease of COVID-19 [7]. The patient was continued on vancomycin and cefepime for possible superimposed bacterial pneumonia. Notably, he had multiple episodes of hypotension with systolic pressure in the high 80s (mmHg) to 90s with mean arterial pressure (MAP) in the low 60s. We administered 500 ml of lactated Ringer’s solution for volume resuscitation. However, aggressive fluid resuscitation was avoided to prevent volume overload. Oral fluids were encouraged to maintain normal volume status. For his COVID-19, as there were no proven specific therapeutic options, the treatment was mainly supportive. He was deemed not a candidate for the clinical trial with remdesivir due to his ESRD. Corticosteroids were not given at that time for concerns that they may delay viral clearance and prolong the disease course [8].

Hemodialysis resumed at the bedside as per the CDC guidelines on hospital day 3 [9]. He remained afebrile, and his blood pressure improved with midodrine and albumin after the dialysis.

Since hospital day 4, the patient started to feel better with increased appetite and improvement of diarrhea, cough, and shortness of breath. Blood cultures demonstrated no bacterial growth at 5 days. With a multidisciplinary approach and optimal medical management, he continues to show clinical improvement despite his comorbidities.

To optimize the use of personal protective equipment (PPE), the patient’s dialysis schedule was changed to Tuesday, Thursday, and Saturday to decrease the dialysis days during his hospitalization. His volume status was closely monitored, as inadequate oral fluid intake and diarrhea could lead to hypovolemia and delay dialysis.

The patient was discharged home on hospital day 9 based on a test-based strategy as per CDC guidelines because of his history of solid organ transplant [10]. No outpatient dialysis precautions were recommended to the patient as he had two negative COVID-19 tests (≥24 h apart) [10].

## Discussion and conclusion

We presented a case of co-infection of influenza A and SARS-CoV-2 in a patient on hemodialysis with no known direct exposure to COVID-19. The patient presented with non-specific symptoms that were clinically indistinguishable from illnesses caused by other respiratory pathogens, and the rapid antigen tests in the ED confirmed influenza A infection. He lived alone and independently at home in western Iowa, and had no recent travel history or visits to any healthcare facilities other than a local dialysis center three times a week for routine dialysis. At the time of his presentation, there was only one confirmed, travel-related case in the county where the patient lives, and the patient denied any direct contact with the first infected person. Although there was no community spread case in the county where the patient lives, community transmitted cases were reported both in western Iowa and Omaha, Nebraska, at the time of this patient’s presentation. Given the increase in the rapid community transmission of the COVID-19 pandemic, our infectious disease team recommended that concomitant infection of COVID-19 needed to be ruled out in this high-risk patient with multiple comorbidities even when there was a lack of clear history of exposure and his symptoms could be easily explained by his positive rapid influenza antigen test. The local health department was notified promptly of the positive COVID-19 results for the investigation of the source of infection. On March 21, 2020, this case was declared as the second confirmed case and the first confirmed community transmitted case in the county [11].

The severity of clinical presentations of COVID-19 ranges widely from asymptomatic, mild to severe illnesses requiring hospitalization and intensive care [6]. As many infected persons remain asymptomatic or only develop mild symptoms, many cases go unidentified and unreported. In a recently published article that studied 16 cohorts worldwide, it is estimated that asymptomatic cases could account for up to 45% of all SARS-CoV-2 infections [12]. The role of asymptomatic transmission in the rapid spreading of the virus is still under investigation, but the same study points out that SARS-CoV-2 can be transmitted from asymptomatic persons to others for a prolonged period.

Around 14% of all infected cases worldwide progress to severe illness and approximately 5% require critical care [6]. Older adults (age > 65 years) and patients with underlying medical conditions such as heart disease, lung disease, diabetes, and renal disease are at higher risk of developing severe COVID-19 illness [13]. In the United States, the preliminary description of outcomes among patients with COVID-19 indicates that fatality was highest in persons aged ≥85, ranging from 10 to 27%, followed by 3 to 11% among persons aged 65–84 years, 1 to 3% among persons aged 55–64 years, < 1% among persons aged 20–54 years, and no fatalities among persons aged ≤19 years [14].

The most common symptoms of COVID-19 patients are fever, cough, fatigue, and GI symptoms [15, 16]. Our patient initially developed mild fever, cough, and shortness of breath, but on admission (illness day 3) and during hospitalization, his main complaints were fatigue and diarrhea. The patient was unfortunately not eligible for the clinical trial of remdesivir at the Nebraska Medical Center due to his ESRD but had been improving clinically with dialysis and supportive care. Of note, remdesivir was later reported to be able to shorten the recovery time from 15 days to 11 days in hospitalized adult patients [17]. By illness day 4 (hospital day 2), his shortness of breath, dry cough, and abdominal pain have improved. Since illness day 5 (hospital day 3), his severe diarrhea also improved with oral loperamide after C.diff infection was ruled out. Our understanding of the treatment options of COVID-19 has been advancing. The patient did not receive any corticosteroids during his treatment based on the popular hypothesis at that time that corticosteroids could potentially delay viral clearance and worsen patient outcomes [8]. However, a more recent large clinical trial showed that dexamethasone could reduce death in patients suffering from severe respiratory complications of COVID-19 by up to one-third [18].

An early study in February on 8274 close contacts in the Wuhan region where the virus was first discovered showed that 5.8% of COVID-19 patients had other respiratory pathogen co-infections [19]. In another study, the rate of co-infections of COVID-19 with other respiratory pathogens was reported to be as high as 21% [20]. A more recent meta-analysis of 30 studies on a total of 3834 patients reports the pooled proportions of bacterial and viral co-infections in COVID-19 patients were 7 and 3%, respectively [21]. Of note, the prevalence of bacterial co-infection was found to be higher in patients in the Intensive Care Unit (ICU), increasing to 14%. Given the increase in the rapid community transmission of the pandemic, the threshold for suspicion and testing for SARS-CoV-2 infection should be low in patients with fever, respiratory symptoms, or other symptoms that are fitting for COVID-19, even when there is evidence of infections by other respiratory pathogens. Any missed COVID-19 cases can lead to significant adverse public health consequences. This case highlights the fact that co-infection of SARS-CoV-2 and other respiratory pathogens can occur in patients with no known direct COVID-19 exposure, and that it is important to test for COVID-19 in high-risk patients even when other etiologies could explain the symptoms [22].

### Lessons learned from this case

1. There should be a high index of suspicion for SARS-CoV-2 infection in patients with fever and respiratory symptoms even when the patient is tested positive for other respiratory pathogens. Any missed COVID-19 cases will put the patient, other patients, health care providers, and the whole community at risk. A high index of suspicion, early testing, and a multidisciplinary approach to patient care are crucial for the treatment of COVID-19 pneumonia, especially in patients with multiple comorbidities.
2. With adequate testing and appropriate triaging of patients with suspected COVID-19, health care providers can be better-prepared in various health care settings, including imaging centers, dialysis facilities, and long-term care institutions.
3. With the widespread community transmission of COVID-19 in the US, patients presenting with other less typical symptoms including GI symptoms such as nausea, vomiting, diarrhea, and abdominal pain should also raise the clinicians’ suspicion for possible COVID-19 infection.

## Data Availability

Not applicable.

## Abbreviations

ESRD: End-stage renal disease
COVID-19: Coronavirus Disease 2019
WHO: World Health Organization
SARS-CoV-2: Severe Acute Respiratory Syndrome Coronavirus 2
US: The United States
WBC: White Blood Cell
CT: Computed Tomography
AV: Arteriovenous
ED: Emergency Department
RT-PCR: Real-Time Reverse-Transcriptase Polymerase Chain Reaction
CDC: Center for Disease Control and Prevention
GI: Gastrointestinal
MAP: Mean Arterial Pressure
PPE: Personal Protective Equipment
ICU: Intensive Care Unit
